# Optimization design of international talent training model based on big data system

**DOI:** 10.3389/fpsyg.2022.949611

**Published:** 2022-07-22

**Authors:** Jing Wang

**Affiliations:** Department of International Communication and Cooperation, Zhejiang Yuexiu University, Zhejiang, China

**Keywords:** big data system, international education, talent training, model optimization, AHP, international talent training model

## Abstract

With the deepening of the concept of a “global village,” the exchanges between countries in the world are deepening. This has led to ever-closer ties between countries and regions. They are more and more interdependent and mutually restrictive. Internationalization has become an inevitable trend and trend in the development of higher education in the process of globalization. Big data refer to a collection of data whose content cannot be captured, managed, and processed by conventional software tools within a certain period of time, and need to be processed to obtain the required information. Developed countries have developed earlier in the field of education internationalization and have more mature experience, which provides a reference for the development of other countries. This paper aims to study the optimization design of the international talent training model based on the big data system. In this paper, the system analysis method is used for theoretical discussion. It establishes an index system of graduate students’ international talent training mode through AHP, and puts forward a breakthrough for international education in colleges and universities. Internationalized talents refer to high-level talents who have an international awareness and mind, a world-class knowledge structure, a vision and ability reaching an international level, and who are good at seizing opportunities and striving for initiative in global competition. This paper deeply studies the current situation of the international talent training model in colleges and universities, and finds out the deficiencies in the international talent training model. It also optimizes the path for the cultivation and development of international talents, which provides a basis for regional development and cultivation of international talents. The experimental results of this paper show that 20.1% of students believe that international education is to adapt to educational development, 25.4% of students believe that it is mainly to meet the needs of student development, and 62.2% of students believe that college majors lack internationalization characteristics.

## Introduction

Talent is the core element of modern country construction, it provides intellectual support for the country’s development. With the advent of globalization and economic integration, the competition between countries has gradually developed into a contest between senior professional international talents with international vision and international knowledge background. College students are the reserve army of talents, so it is imperative to carry out international education for college students. The cultivation of international talents needs to involve big data technology. The big data model research on the quality evaluation of talent training in colleges and universities is mainly reflected in the evaluation, which can show the level of talents as a whole. China has a relatively short development time in international education personnel training, so it needs to learn from the advanced experience of other countries (Hong et al., 2019). This paper aims to study the optimization design of the international talent training model based on the big data system. Through the cultivation of international talents, it is conducive to realize the effective supply to the labor market and solve the problem of insufficient supply of high-quality talents in the labor market. This establishes an internationalized training goal that meets the development characteristics of colleges and universities and meets the requirements of economic and social development, and puts forward relatively complete countermeasures and suggestions for the training mode of internationalized talents in higher vocational colleges. It cultivates general-purpose technical talents with international competitiveness in accordance with the needs of the international industry, which provides an internal driving force for the stable and rapid development of the local economy.

In order to improve the comprehensive quality of students and cultivate versatile talents with practical ability, education needs to combine big data technology and innovative training models. On the basis of the research on Russian talent training and the use of big data technology, Huang Y proposed a multidisciplinary talent training model featuring culture, language, and interaction. Through the application of the CLI model, optimizing the curriculum system and teaching methods, and building a good learning environment, traditional talent training has been greatly improved (Chau et al., 2021). Big data technology can store huge amounts of data. It can capture and collect various types of data. Big data analysis has high commercial value and application value and fast calculation speed. Under the current situation of poor targeting and unreasonable allocation of class hours for international Russian majors, the new training model of CLI has had a positive impact on the training of international Russian talents in higher vocational colleges (Li et al., 2021). Hotel managers play an important role in hotel operations and operations. For a long time, researchers have focused on selecting outstanding hotel management talents. Nowadays, with the development of China’s economy and the rapid development of the hotel industry, the demand for management talents is also increasing. Although researchers have established a variety of talent selection and development models after analyzing a large amount of data on hotel management majors in domestic universities, these models are still far from development needs. Therefore, in response to the academic neglect of the theoretical research on the selection of motivational talents, Xu Q proposed a selection scheme for motivational hotel management talents. He made a fuzzy comprehensive evaluation on the satisfaction of hotel managers’ development process by establishing a hotel manager gap measurement model, questionnaire survey, and sample data collection. He found the hotel manager selection process to be more efficient and effective. The fuzzy comprehensive evaluation results of the talent development process are consistent with the real-life sample statistics. Finally, he put forward optimization suggestions based on the conclusions, including the cooperation between hotels and universities and the utilization of resources inside and outside the school. Fuzzy comprehensive evaluation has the characteristics of clear results and strong systematicness. It can better solve vague and difficult to quantify problems, and is suitable for solving various non-deterministic problems. It is necessary to strengthen the teaching of practical skills in colleges and universities and establish a development mechanism for hotel management talents, which meets the needs of hotel industry talents (Ye and Wang, 2021). Ma H aimed to improve the quality and efficiency of talent training in colleges and universities, and effectively analyze and count the number of talents. It takes the training of computer professionals in higher vocational education as the research object. First of all, it uses deep learning neural network and data mining algorithm, introduces recurrent neural network (RNN) and fusion attention model, and develops a college talent training information system. According to the needs of employment forms and employment data, it formulates a diversified talent demand training program. On this basis, it establishes a set of efficient talent training quality index system through the analysis of higher vocational education talent training data. Finally, the effectiveness of the system is verified by performance analysis. The results show that the model is not only feasible for the classification of computer professional personnel training, but also can accurately reflect the problems in personnel training, and the results are visualized (Ma and Ding, 2021). Although these theories describe international education, they do not introduce how to develop international education.

The concept of big data is now approached from a different perspective, covering its impact in many fields, including healthcare. For a wealth of health information, Bikash began by providing the lay reader with a broad overview of the effectiveness of big data and big data in healthcare. He then built a distributed framework for an organized healthcare model to protect patient data (Sarkar, 2017). Money W H analyzed the nature of unknown failures in big data systems for knowledge management and real-time processing of big data. These errors create risk and threaten the knowledge pyramid and decision-making based on knowledge gleaned from large volumes of complex data. He hypothesized that failures not yet encountered may require failure handling, analytical models, and architectural frameworks to assess and manage failures and reduce the risk of correlating or integrating otherwise unrelated big data. He also ensured the source lineage, quality, integrity, freshness, and validity of the collection data. The design of the system must mitigate failures caused by real-time stream processing, while ensuring that variables such as synchronization, redundancy, and latency are addressed. Experimental results show that, with improved design, real-time big data systems may continue to provide the value of streaming big data (Money and Cohen, 2018). As technology advances, raw sensor data are further calibrated and processed, and additional data products are derived. This greatly reduces the burden of preprocessing these data on downstream applications. Stephen E discussed current open datasets and how these datasets can be used to solve various problems in agriculture. Additionally, he discussed implementing a cloud-based scalable agricultural information system that provides farmers with actionable insights. Deep learning and spatiotemporal data mining algorithms can be applied to these data to extract hidden information. Despite its apparent simplicity, collecting, analyzing, and gaining insights from these sensor data and other data products from numerous sources is a challenge for big data and high-performance computing (Stephen, 2021). Huang J first splits the ecological environment information system, and then conducts a detailed study on the application of the ecological environment information system based on big data technology. Then, he started with the analysis of the characteristics of big data, and analyzed the development of big data in the environmental field. He revealed the database of soil environmental big data development and the difficulties encountered in the development process, and discussed the soil environmental big data system, construction method, and development content. According to the big data development strategy and industry needs in the field of soil environment, he suggested to build a soil environment big data cloud platform to achieve cross-media collaborative management within the region (Huang, 2021). Traditional databases cannot handle unstructured data and massive real-time datasets. Traditional databases are relational databases. The purpose of developing such a database is to process permanent, stable data, but it is difficult to take into account the timing constraints on the data and its processing. This cannot meet the needs of real-time applications in industrial production management. Diverse datasets are unstructured big data. Storing, managing, processing, analyzing, visualizing, and extracting useful insights from these datasets is laborious using traditional database methods. However, under the trend of big data, there are many technical problems in the extraction of large heterogeneous datasets. T aimed to present an overview view of a complete big data system. The system includes several stages of processing big data and key components of each stage. He looked at case studies of distributed ML tools. Additionally, he categorized analytics by data type, domain, and application. Finally, he proposed some key points related to research directions and opportunities based on current trends in big data. Studying big data infrastructure tools with recent developments can lead to a better understanding of how different tools and techniques can be applied to solve real-life applications (Rao et al., 2019). These theories explain big data, but they do not explain the relationship between big data and international education personnel training, and they are not practical.

Human resources are an important force for national development. With the deepening of internationalization, international talents are an important force for national development. The cultivation of large numbers of people is inseparable from education, so the cultivation of international talents needs the support of international education. Research shows that 43.1% of students believe that the mastery of international professional knowledge is insufficient. 75.6% of the students believe that the foreign language level needs to be improved. 52% of students believe that intercultural communication skills are insufficient. 35% of students believe that personal physical and mental quality is an important aspect that affects the cultivation of international education.

## Optimal design method of talent training model

### Cultivation of international talents

As the form of world multi-polarization intensifies, the trend of multi-polarization in both political and economic fields is accelerating worldwide (Xu et al., 2019). From the overall situation, human beings are currently in a stage of peaceful development, and the exchanges and cooperation between countries are getting closer. In order to penetrate into social and economic development and progress, we need international talents to promote cooperation, which requires more international talents to join (Zhang and Wang, 2021). Cultivating international talents is an inevitable requirement of economic globalization. Cultivating international talents is a common choice for all countries to enhance their international competitiveness. At the same time, it is the mission and responsibility of foreign-related universities to cultivate international talents.

Internationalized talents are strategic resources for countries to improve their comprehensive national strength and competitiveness. Therefore, it is more important to cultivate local talents instead of relying only on foreign talents. According to China’s education system, colleges and universities are important places for cultivating international talents (Reuther et al., 2018; Zhou et al., 2022). International talents need humanistic quality and broad knowledge. They need to have strong foreign language application ability and cross-cultural communication ability. They need to have a sense of national responsibility and inherit excellent national culture. They need to have high information literacy and use modern technology to analyze and process information.

**Figure 1:**
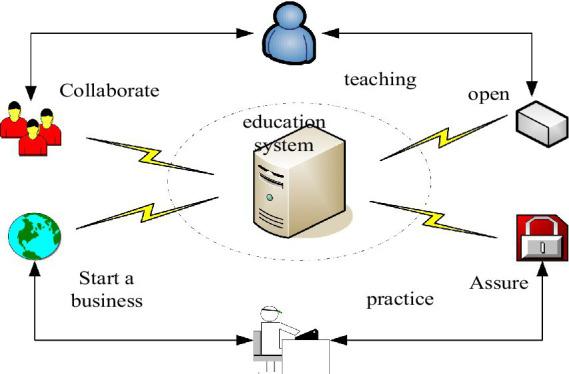
Brief model structure of talent training model.

The term “cultivation model” was first used in the field of education, but there is no academic explanation for it in the relevant literature, so everyone has different opinions on the meaning. However, it is generally agreed that talent training is a systematic and dynamic process (Xu, 2019). Figure 1 shows the brief model structure of the talent training model.

**Figure 2:**
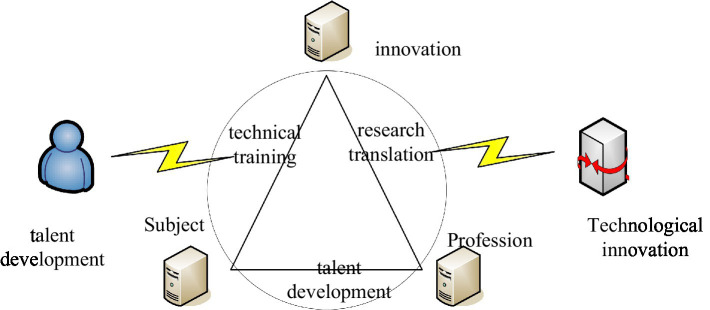
Contents related to the cultivation of modern talents.

Strictly speaking, talent cultivation and talent cultivation mode are different concepts. Patterns are an important method of operation. In layman’s terms, it is a bridge. It closely links theory and practice, and uses theory to guide practice. The talent training model can be regarded as a method of teaching activities, but it does not grasp the essence of talent training (Wei et al., 2019; Ye et al., 2019). The talent training mode refers to a standard education style and education realization method set up under the guidance of scientific education theory to cultivate students’ sound knowledge, ability, and quality system. The most important thing for talent training is to design the composition of the model. As far as its composition is concerned, the training objectives are mainly determined according to social needs, and then majors and courses are set up. However, with the continuous progress of society, people’s requirements for talents are getting higher and higher, and more attention is paid to the comprehensive development of students in the training process (Przednowek et al., 2019). It is mainly used in the division problems involving some numbers that are difficult to be directly obtained by the combination equation. For example, it divides n numbers into multiple division methods that are not greater than m, and there is no same number, or the problem of dividing them into several odd or even numbers. Figure 2 shows the content related to the cultivation of modern talents.

### Data processing

With the advancement of science and technology, we need more and more information. With the promotion of the Internet, there are more and more data and information around us, which brings difficulties to the use of information. In order to use the information reasonably, we need to classify various types of data information (Gilani and Sahebi, 2021; Wu et al., 2022).

**Figure 3:**
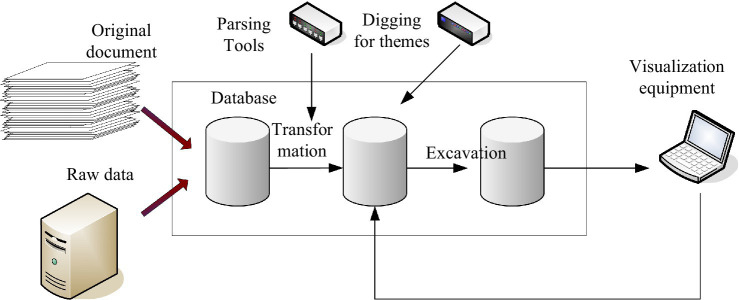
Architectural model of the data processing system.

Clustering refers to the process of classifying mined data. It can integrate information, analyze data centrally, and maximize the value of data. Figure 3 shows the architecture model of the data processing system.


(1) $$\left(g_{1},g_{2},\cdots g_{k}\right)$$

Equation (1) represents a data matrix, in which each row in each matrix represents a data object. It can be seen that each matrix has an attribute.


(2) $$b_{op}=\frac{b_{op}-{\bar{b}}_{op}}{\varepsilon_{p}}$$

(3) $$b_{o}=\frac{2}{k}\sum b_{op}$$

In the above equation, $b_{op}$ represents the common data specification, and Equation (3) represents the mean of the data.


(4) $$\varepsilon_{p}=\sqrt{\frac{2}{k-2}\sum \left(b_{op}-{\bar{b}}_{p}\right)}$$

Equation (4) represents the standard deviation of data on different attributes.


(5) $$b_{op}=\frac{b_{op}}{b_{pmax}}$$

Equation (5) represents the maximum value of data on an attribute.


(6) $$b_{op}=\frac{b_{op}}{{\bar{b}}_{p}}$$

Equation (6) represents the normalized mean, which represents the value of the data when the attribute is.

The division algorithm divides the objects in the data set into several parts, and through repeated iterative calculations, the objective function is continuously reduced until convergence. It is mainly used in the division problems involving some numbers that are difficult to be directly obtained by the combination equation. For example, it divides n numbers into multiple division methods that are not greater than m, and there is no same number, or the problem of dividing them into several odd or even numbers.


(7) $$\mathrm{SSE}=\sum \sum t\left(Q_{f}\mathrm{,k}\right)$$

In Equation (7), t(∗) represents the distance between different data objects. Under other conditions being the same, the quality of the results is affected.

**Figure 4:**
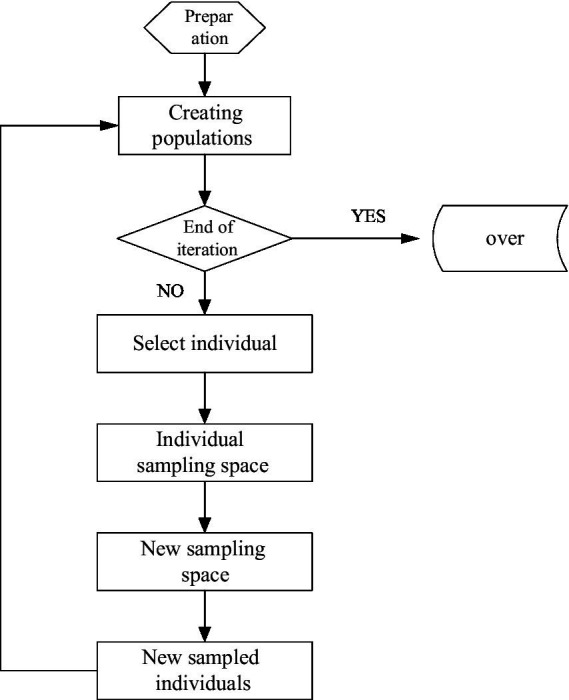
Queue intelligence algorithm flow.

The queue intelligent algorithm is a relatively new intelligent algorithm. Because the algorithm is relatively new and has never been widely used in various fields, it is also widely used in the processing of clustering problems. Its algorithm flow is shown in Figure 4.

In this cluster analysis, it is not carried out in every generation of evolution, and there are several intervals between them. This method conforms to the operation law of the algorithm, which is beneficial for the algorithm to use the information accumulated iteratively, and conforms to the overall process and trend of intelligent algorithm optimization.


(8) $$\mathrm{cutoff}=3-\frac{\mathrm{YU}}{\mathrm{YR}}$$

In Equation (8), YU represents the number of times to perform hierarchical clustering on the data, and YR represents the number of times that should be done in the evolutionary process.


(9) $$\mathrm{YR}=\frac{\mathrm{KH}}{\mathrm{FB}}$$

In Equation (9), KH represents the maximum evolutionary algebra, and FB represents the clustering interval algebra for the population.


(10) $$\mathrm{center}=\frac{\sum Q_{ab}}{d},b=1,2,\cdots ,r$$

center represents the component of cluster centers, while d represents the number of cluster centers.


(11) $$\mathrm{distance}\left(t\right)=\sqrt{\sum \sum \left(k_{ab}-q_{ab}\right)}$$

Equation (11) represents the Euclidean distance from all objects to a certain cluster center, t represents the cluster center, and $q_{ab}$ represents the b th attribute value of object q .


(12) sumdistance=∑distance(t)

When all the data are sorted, we can derive the distance represented by each candidate solution. When we combine them together, it can be expressed by the Equation (12).


(13) fitness=2sumdistance

In the above equation, sumdistance is the objective function of the clustered data. When we want to get its minimum distance, we can use Equation (13) to calculate.

When the data are clustered and analyzed, certain results will be obtained, but the results are not necessarily the best values, so we need to evaluate and analyze them.


(14) $$R\left(\mathrm{c,v}\right)=\frac{3y\left(\mathrm{c,v}\right)\theta \left(\mathrm{c,v}\right)}{y\left(\mathrm{c,v}\right)+\theta \left(\mathrm{c,v}\right)}$$

In Equation (14), R is used to evaluate the effect of the clustering problem, where y represents recall and θ represents precision.


(15) $$R=\sum \frac{g}{G}\max\left(R\left(\mathrm{c,v}\right)\right)$$

Equation (15) represents the processing of the clustering problem of a dataset whose scale is *G*.

**Figure 5:**
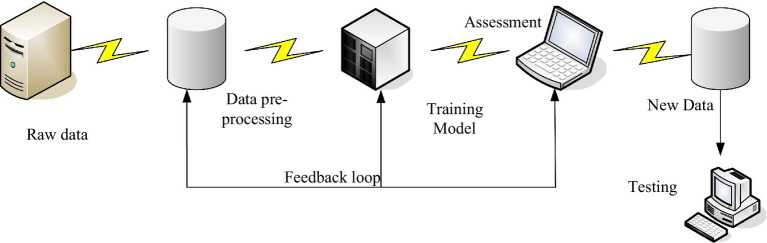
Data normalization processing structure.

In actual processing situations, the amount of data is very large. In order to reduce the influence of the data volume on the clustering effect, we need to normalize the data. Figure 5 shows the structure of data standardization processing.


(16) $$T_{uo}=\frac{T_{uo-}\bar{T_{u}}}{Q_{u}}$$

(17) $$\bar{T_{u}}=\frac{2}{k}\sum T_{uo}$$

(18) $$Q_{u}=\sqrt{\frac{2}{k}\sum \left(T_{uo}-\bar{T_{u}}\right)}$$

The above equation translates the data. However, the data are still not standard data at this time, and translation range transformation is still required at this time.

### International education

**Figure 6:**
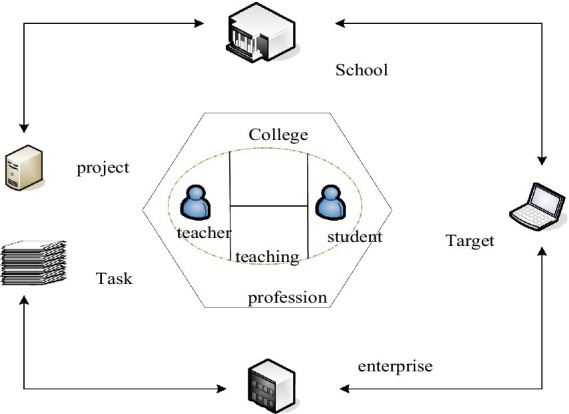
Framework of an international education model.

In the context of economic globalization, the society’s demand for international talents is getting higher and higher. The internationalization of talents means the internationalization of education. Internationalization of education refers to the integration of local courses with international courses, international exchanges between teachers and students, and technical assistance between cross-border schools. Here, we mainly discuss the internationalization of higher education. Internationalization of higher education is not an end, but a means to cultivate a world-minded, high quality, and ability. From the perspective of historical origin, the internationalization of higher education can be traced back to the reform of the educational system in ancient Greece. Through its continuous exploration and improvement, it enables practice to have theoretical support and improves the quality of teaching and education. The internationalization of early higher education is an individual behavior, which is mainly for the pursuit of knowledge, while the internationalization of modern higher education is more to promote national development and enhance one’s own ability (Latukha, 2018; North et al., 2021). Figure 6 shows the framework of an international education model.

With the increasing development of economic globalization, the exchanges and cooperation of higher education have gradually increased, and its internationalization trend has become stronger and stronger. It has changed from “government-led” to “college-led.” Its forms and means are increasingly diverse. The competition in the market is becoming more and more intense. Internationalization of higher education is a process from theory to practice. It learns from the experience of other schools with higher education levels and introduces advanced training concepts and goals. At the same time, the internationalization of education development also provides the possibility for the sharing of educational resources.

## International talent training experiment

### Overview of the basic situation of the experiment

As China’s communication with the world becomes more and more close, it is also more and more in line with international standards in the cultivation of talents. The current talent is an important force for the development of the country, and it is very important to the cultivation of the number of people. From the perspective of the current education system, the most important thing about how to integrate talent training with internationalization is the education method of colleges and universities. For this reason, we conducted a brief survey of the current education system in colleges and universities, as follows.

According to the data in Table 1, we have introduced the basic objects of the experiment. Based on the data collected, we surveyed 200 students in colleges and universities. Among them, 134 were boys, accounting for 67.1%, and 66 were girls, accounting for 32.9%. According to the current gender ratio of students, it can be seen that this combination of conditions is more reasonable, and it can reflect the real situation. Secondly, we organize the data recovery. Among them, the effective recovery rate of boys was 88.6%, and the effective recovery rate of girls was 90.3%. According to the recovery situation, the recovery situation of the two groups of data is relatively high. This shows that the subjects participated in the questionnaire relatively high, which is helpful for us to investigate the cultivation of international talents in colleges and universities (Table 2).

**Table 1:** Basic introduction of the survey.

Category	Male	Female
Number of people	134	66
Proportion	67.1	32.9
Effective recovery	88.6	90.3

**Table 2:** Basic information of survey content questions.

Category	Quantity	Proportion
Training overview	5	23.8
Model evaluation	3	14.3
Curriculum design	3	14.3
Facility input	3	14.3
Cooperation and exchange	3	14.3

Questionnaire surveys can collect data as much as possible, especially electronic questionnaires, which can improve the efficiency of data collection. In order to play its role better, the problem should be designed more rationally and scientifically. In order to investigate the international training situation of colleges and universities, we listed 21 questions. The first 4 of them belong to the basic information of the participants, and we will not introduce them here. Among the questions in the questionnaire, we set 5 questions about the cultivation profile, which reached 23.8%. In addition, we also conducted investigations from the aspects of model evaluation, curriculum design, facility investment, and cooperation and exchanges. It sets up three questions in these four areas, which are 14.3%. From the introduction of the questionnaire, it can be seen that the questions revolve around international talents. It can understand the basic situation of colleges and universities, and at the same time, it can understand students’ views on the system, which provides a basis for our understanding of their training models.

### Evaluation and analysis of talent training mode

When introducing the dimensions of the questionnaire, we pointed out the evaluation settings of the talent training model. This question can reflect the students’ basic attitude towards the school setting. We can analyze the unreasonable aspects of the system and make reasonable adjustments according to the students’ attitudes. The talent training in this university improves the theoretical basis and improves the internationalization level of students. For this reason, we conducted a survey on the evaluation of colleges and universities, as follows.

According to the data in Table 3, we have conducted a survey on the attitudes of students based on the cultivation of talents in colleges and universities. According to the data, there are 50 people who think schools are relatively neglected, reaching 24.8%. 86 people think that the school attaches great importance to them in general, reaching 43.2%. There are 53 people who think that the school attaches more importance to it, reaching 26.3%. 12 people think that the school attaches great importance to it, reaching 5.7%. According to the survey, more than two-thirds of the students believe that the school lacks attention to the cultivation of international talents. In fact, colleges and universities have always believed that the cultivation of applied talents is the key point, and the whole society is skeptical about the internationalization of talents in college education. These external reasons have hit the enthusiasm of colleges and universities to cultivate talents. All the concepts of international talent training cannot be thoroughly implemented in the education concept, which leads to this relatively embarrassing situation.

**Table 3:** Basic situation of international talent cultivation concept in universities.

Category	Quantity	Proportion
Comparative neglect	50	24.8
Generally	86	43.2
More important	53	26.3
Very important	12	5.7

### Investigation and analysis of facility investment

As can be seen from the foregoing, in terms of setting up the questionnaire, we have investigated the basic investment in international facilities. For the investment in basic facilities, we mainly discuss the equipment of the laboratory. Based on this, we can see the hardware investment of colleges and universities in talent training.

According to the data in Table 4, we have investigated the laboratory configuration of different majors and divided them into four categories. From the above data, 87 people indicated that the basic facilities of the laboratory have been equipped, reaching 43.7%. 46 people said that the basic facilities of the laboratory are being equipped, reaching 23.1%. 21 people said that the basic facilities of the laboratory are planned to be equipped, reaching 10.3%. 45 people indicated that the basic facilities of the laboratory were not equipped, accounting for 22.9%. It can be seen that the basic equipment of laboratories in colleges and universities has begun, but it has not been completed at present, and it is still in the development process.

**Table 4:** Survey of professional settings and laboratory equipment.

Category	Quantity	Proportion
Set up	87	43.7
Being set up	46	23.1
Planning to set	21	10.3
No	45	22.9

## Optimization analysis of international talent training model

### International education

International education can drive students to develop in a more comprehensive direction and to integrate with internationalization more quickly. At present, colleges and universities are still in the initial stage in the cultivation of international education, and their understanding of international education is not deep enough. In order to investigate students’ understanding of international education, we conducted a survey and analysis accordingly.

**Figure 7:**
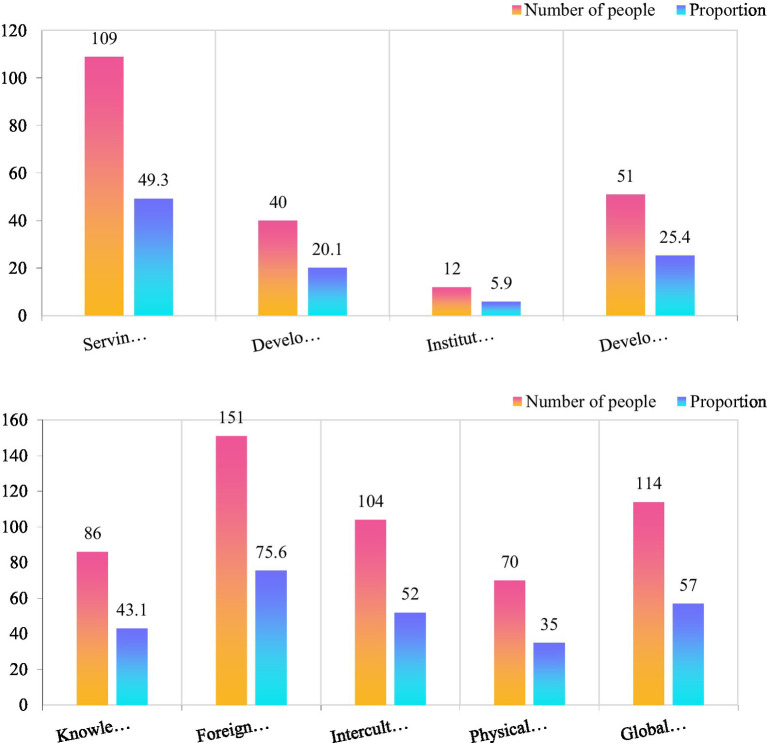
Awareness and deficiencies of international education.

International education needs to serve social development. According to the data in Figure 7, we have launched a survey on the understanding of international education. According to the above data, 49.3% of the students believe that the implementation of international education can better serve the society, and the proportion is nearly half. 20.1% of the students believed that the promotion of international education is to adapt to the development of education. 5.9% of students believe that the promotion of international education is to improve the international competitiveness of colleges and universities. 25.4% of students believe that the implementation of international education is to meet the needs of students’ development (Ivanaj et al., 2019). It can be seen that in the students’ minds, they subconsciously believe that internationalization is the future development trend, and international talents are an important resource for promoting social progress. This kind of thinking is very practical and helps to inspire students to develop in a more scientific direction.

Judging from the weakness of college students in international education, 43.1% of the students believed that the mastery of international professional knowledge was insufficient. Professional knowledge is the basic knowledge that talents need to master, and this aspect needs to be strengthened. 75.6% of the students believe that the foreign language level needs to be improved. Foreign language is the basic way of international communication, and it is a communication skill that must be mastered. 52% of students believe that the mastery of intercultural communication is incomplete. Cross-cultural communication is a necessary ability for international talents and needs to be cultivated slowly. 35% of students believe that personal physical and mental quality is an important aspect that affects the cultivation of international education. 57% of students believe that there is a lack of global awareness in international education. According to the above data, foreign language proficiency is the most in need of improvement in international training, followed by a global vision and cross-cultural communication. From this, it can be seen that students basically understand the standards of international talents, and accurately realize which abilities need to be cultivated.

**Figure 8:**
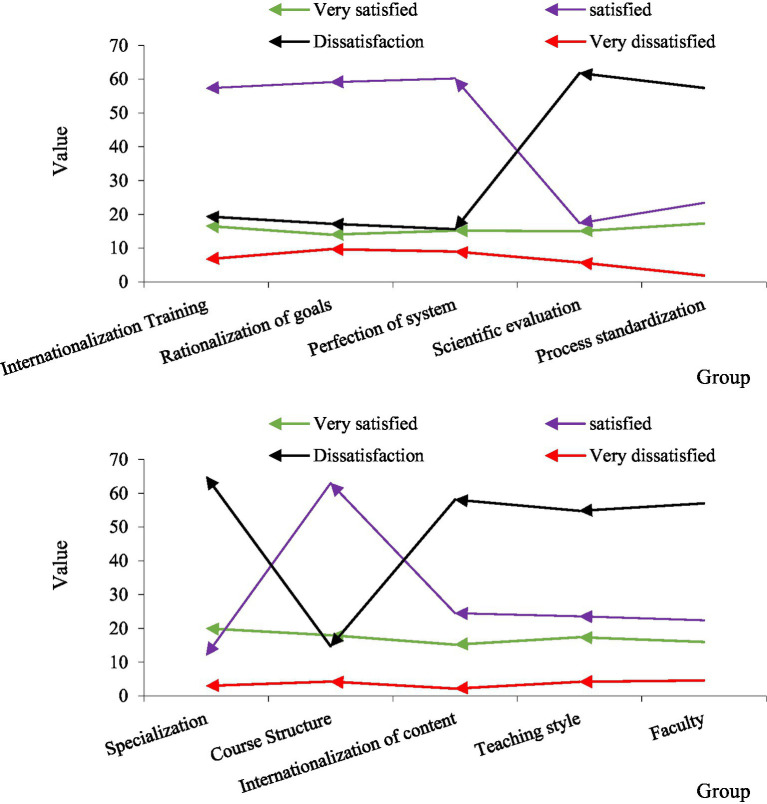
Talent training satisfaction analysis.

In order to explore students’ satisfaction with the cultivation of international talents, we conducted a detailed survey on this dimension. According to the data in Figure 8, we have explored the satisfaction of the talent training mode and process, respectively. First of all, from the perspective of talent training mode, in terms of scientificity, more than two-thirds of the students are dissatisfied. In terms of normativeness, more than 60% of students are dissatisfied. From the other three dimensions, satisfaction is generally high, which shows that the system and goals are more in line with the development law of students. It can be seen that the international talent training model of colleges and universities is generally good, but needs to be improved in terms of evaluation and standardization.

Judging from the satisfaction of the talent training process, 65% of the students think that the professional setting is unreasonable, and 58.1% of the students are dissatisfied with the teaching content, 54.8% of the students were dissatisfied with the teaching methods, and 57% of the students were dissatisfied with the teachers in colleges and universities. Most students believe that the course structure and methods are more reasonable and more in line with the needs of students. It can be seen from this that colleges and universities pay more attention to form than practice in international training, and there is still a large distance between the actual teaching link and communicative education.

### Major and curriculum design

Courses are an important source of knowledge for students, so the design of courses is very important for students. In order to analyze the problems of colleges and universities in curriculum design, we conducted a brief survey of students’ attitudes, as follows.

**Figure 9:**
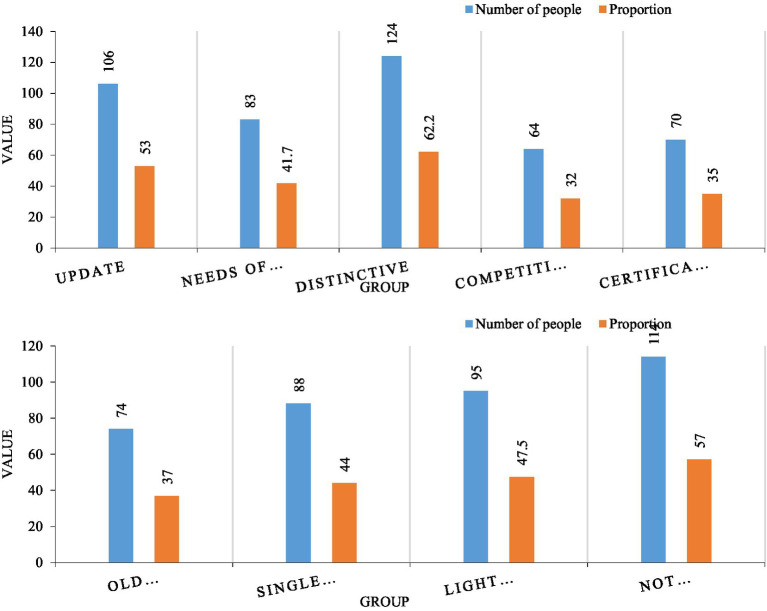
Professional and course design analysis.

In order to analyze the curriculum design of colleges and universities, we analyze the problems and implementation obstacles of curriculum design. First of all, we need to look at the problems existing in the curriculum design. According to the data in Figure 9, the professional setting of 106 students cannot keep up with the development of society, reaching 53%. It can be seen that colleges and universities are relatively slow in updating their majors, and more than half of the students have this view, indicating that the problem is relatively serious. 124 students believed that the majors of colleges and universities lacked international characteristics, reaching 62.2%. These two problems are very prominent in the curriculum setting, which shows from the side that the current colleges and universities lack experience in the cultivation of international education. In addition, it is worth paying attention to the demand for talent structure. 41.7% of students believe that the current professional setting is not in line with the current social needs. This model is not only a waste of educational resources, but also inconsistent with the frequency of social development.

From the perspective of the promotion of internationalized courses, 88 people believed that the unreasonable course setting was caused by the single course structure, accounting for 44%. 114 people think that the curriculum is unreasonable because the curriculum lacks practicality and is difficult to really use. 57% of the students in this group, and 47.5% of the students thought that disdain for the basic courses of international education was the reason why the courses were unreasonable. It can be seen from this that there are few practical courses in international education and training, and it is impossible to transform theory into practice.

### Communication and cooperation

To cultivate students with internationalization ability, it is not only necessary to design the courses, but also to carry out international exchanges and cooperation in the learning process, so as to cultivate students’ foreign communication ability. At the same time, the international cooperation and exchange activities of colleges and universities are also an important basis for reflecting the internationalization level of colleges and universities. In order to explore the internationalization level of colleges and universities, we conducted a survey on the international exchange activities of colleges and universities. The details are as follows.

**Figure 10:**
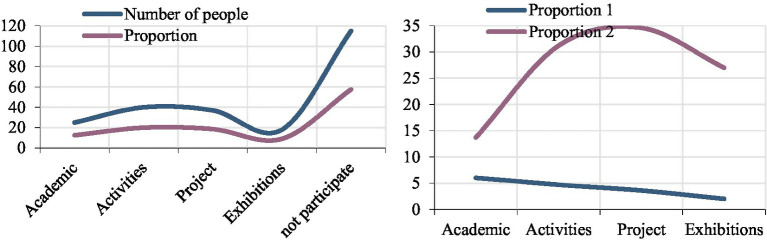
Analysis of international exchange and cooperation.

In order to explore the internationalization capabilities of colleges and universities, we conducted a survey on the local exchange forms and numbers of colleges and universities. According to the data in Figure 10, we first investigated the activity form. According to the statistics, 20% of the international students have social activities. It was the highest option among surveyed projects, closely followed by overseas project exchanges. The two groups have a higher proportion of activities, but on the whole, the proportion of all projects is relatively low, indicating that there are fewer forms of international exchanges in colleges and universities. In addition, it is worth noting that the number of people who have not participated in international exchange activities is very large, indicating that the overall level of internationalization of colleges and universities is low.

Judging from the number of international exchanges, basically every project has people participating, which shows that the international cooperation and exchange activities of the colleges and universities are still in progress. However, international school cooperation and exchanges are mainly short-term activities, and these activities have restrictions on the number of students, and many students cannot participate in them, resulting in a low overall level of internationalization. Schools should organize activities that can accommodate multi-person exchanges to meet the developmental needs of students.

## Conclusion

The wave of economic globalization makes international exchanges more and more closely, and international needs international talents to promote. This requires the country to cultivate international talents. The cultivation of talents is inseparable from education, so colleges and universities have a great responsibility for the cultivation of international talents. The most fundamental thing to establish an internationalized talent training concept is to have a correct understanding and accurate positioning of the value of internationalization. Therefore, this paper aims to study the optimization design of the international talent training model based on the big data system. It expects to use big data technology to provide technical support for the cultivation of international talents and promote the development of education in a more comprehensive direction. In the process of exploration, it is found that the current international education in colleges and universities needs to be improved. Especially in terms of curriculum design and professional setting, it is not at the same frequency as the current development of society. The curriculum lacks practice, and it fails to translate theory. Although this paper has drawn some conclusions, there are still some shortcomings. In the process of using the big data evaluation model to conduct research in this study, although it has been studied, due to personal background constraints, it lacks a broad perspective and the analysis is relatively shallow. It hoped that subsequent studies could explore from a deeper perspective.

## Data availability statement

The original contributions presented in the study are included in the article/supplementary material, further inquiries can be directed to the corresponding author.

## Author contributions

JW: writing—original draft preparation, editing, data curation, and supervision.

## Funding

This work was supported by Key Project of Zhejiang Yuexiu University: Research on Internationalization Strategy of Foreign Language Universities under the Background of New Liberal Arts (Project no: D2020007).

## Conflict of interest

The author declares that the research was conducted in the absence of any commercial or financial relationships that could be construed as a potential conflict of interest.

## Publisher’s note

All claims expressed in this article are solely those of the authors and do not necessarily represent those of their affiliated organizations, or those of the publisher, the editors and the reviewers. Any product that may be evaluated in this article, or claim that may be made by its manufacturer, is not guaranteed or endorsed by the publisher.
